# 

**Published:** 1854-10

**Authors:** 


### CONTENTS OF THIS (OCTOBER) NUMBER.

ORIGINAL COMMUNICATIONS.

Method of Directing Second Dentition. By James Taylor M. D., D. D. S............. 1
American Society of Dental Surgeons................................................ 9

SELECTED ARTICLES.

A Dissertation on the Diseases of the Dental Pulp and their Treatment. By Chapin A. Harris, M. D., D. D. S................................................ 11
Hygienics of Temperance, or Water and Alcohol Contrasted on the People Proper. By Samuel A. Cartwright, M. D................................................ 27
Dental Education By J. S. Rock, M. D., D. D. S.............................. 34
Rattlesnakes and Delirium Tremens.............................................. 39
Biographical Notice of the late Josiah Foster Flagg, M. D., D. D. S.................. 42
Dental Patents............................................................... 49
Dental Abstracts............................................................. 54
Editorial.................................................................... 73

